# A randomized, controlled non-inferiority trial comparing A(H1N1)pmd09 vaccine antigen, with and without AS03 adjuvant system, co-administered or sequentially administered with an inactivated trivalent seasonal influenza vaccine

**DOI:** 10.1186/1471-2334-12-279

**Published:** 2012-10-30

**Authors:** Joanne M Langley, Louise Frenette, Laurence Chu, Shelly McNeil, Scott Halperin, Ping Li, David Vaughn

**Affiliations:** 1 Canadian Center for Vaccinology, IWK Health Centre, Capital District Health Authority and Dalhousie University, 5850 University Avenue, Halifax, NS, B3K 6R8, Canada; 2 Q&T Research Inc, Sherbrooke, QC, Canada; 3 Benchmark Research, Austin, TX, USA; 4 GlaxoSmithKline Vaccines, King of Prussia, Philadelphia, PA, USA

**Keywords:** Adjuvant, AS03, Co-administration, H1N1, Influenza, Pandemic, TIV

## Abstract

**Background:**

At the time of the influenza A(H1N1)pmd09 pandemic it was not known if concurrent or sequential administration of seasonal trivalent influenza vaccine (TIV) with pandemic vaccine was preferred.

**Methods:**

Immunogenicity and safety were assessed in 871 healthy subjects aged 19–40 years who were randomised into six groups to receive co-administration or sequential administration of TIV and two doses of A(H1N1)pmd09 vaccine (either unadjuvanted or adjuvanted with AS03, an α-tocopherol and squalene-based oil-in-water emulsion).

**Results:**

Safety and immunogenicity data (by haemagglutination inhibition [HI] assay) after each dose and six months post-Dose 1 are reported here. Co-administration of A(H1N1)pmd09 vaccine with TIV reduced the HI immune responses to A(H1N1)pmd09 vaccine. However, serologic responses with both co-administration and sequential schedules met the European and US regulatory criteria for pandemic and seasonal influenza vaccines up to six months following the first vaccine dose. The AS03-adjuvanted formulation elicited higher immune responses at all time points. Prior administration or co-administration of A(H1N1)pmd09 vaccine did not affect immune responses to TIV.

**Conclusions:**

Co-administration of TIV and A(H1N1)pmd09 vaccine negatively influenced A(H1N1)pmd09 vaccine immunogenicity but had no effect on TIV responses. The non-adjuvanted and adjuvanted vaccines demonstrated strong immune responses against all vaccine strains for up to six months following the first vaccine dose.

**Trial registration:**

NCT00985673

## Background

In contrast to seasonal influenza outbreaks, high attack rates and substantive morbidity during the influenza A(H1N1)pdm09 pandemic appeared in young persons and adults under 65 years of age [1], and in subsequent analyses more than 75% of A(H1N1)pmd09-related deaths in the US were estimated to have occurred in those aged 18–64 years [2]. Immunisation is considered to be an essential component of public health strategies to mitigate both seasonal and pandemic influenza illness and mortality. As the first wave of the A(H1N1)pmd09 pandemic passed, and the second wave began in the North American fall of that year, it was not known whether seasonal trivalent inactivated influenza vaccine (TIV) should be given before, after, or concurrently with the pandemic vaccine, or at all, during a pandemic year. Early results from a National Institute of Allergy and Infectious Diseases (NIAID) trial in 400 healthy adults published online in October 2009 suggested that co-administration of the two vaccines did not impair immune response to either vaccine [3], but otherwise there was little or no direct data on the immunogenicity and safety of co-administration of TIV and pandemic influenza vaccines to guide decision making.

In addition to the potential safety and immunogenicity considerations of concurrent or sequential administration of TIV with a novel influenza vaccine antigen, it was not known if pandemic vaccines using an oil-in-water adjuvant would alter the immune response to TIV given before, concurrently, or after the pandemic vaccine. The World Health Organization (WHO) recommended production and use of oil-in water adjuvants (and live attenuated influenza vaccines), based on an anticipated limited vaccine availability on a global level [4].

In this study the safety and immune responses to A(H1N1)pmd09 pandemic vaccine, with or without AS03 adjuvant (an α-tocopherol and squalene-based oil-in-water emulsion adjuvant system), following co-administration or sequential administration of TIV, was evaluated in young adults. Although serologic responses met regulatory criteria for approval of such vaccines, co-administration was associated with reduced A(H1N1)pmd09 vaccine immunogenicity. No effect on TIV immunogenicity was observed, and both non-adjuvanted and adjuvanted vaccines demonstrated strong immune responses against all vaccine strains for up to six months following the first vaccine dose.

## Results

### Participant flow

Of the 871 subjects enrolled and screened, 611 subjects were vaccinated; 580 and 561 subjects completed Day 63 and Day 182 visits, respectively. Demographic characteristics in the six study groups (TVC) were similar. The mean age of subjects was 28.8 years (range: 19–40 years). The male to female ratio was 45.2%:54.8% and the majority of subjects (79.2%) were of European/Caucasian ancestry.

**Figure 1:**
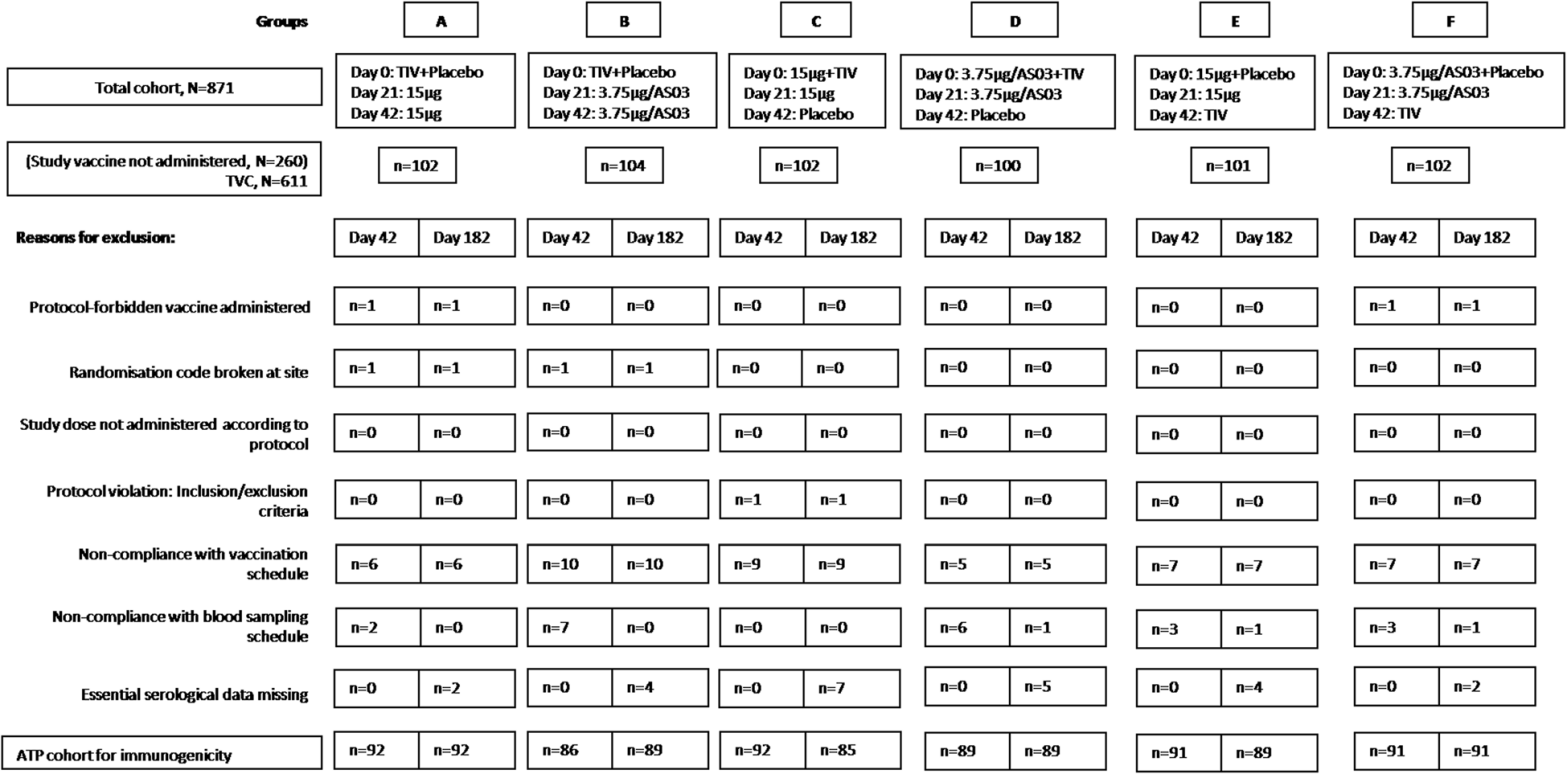
**Study design.** Total vaccinated cohort (TVC): all subjects with at least one documented vaccine dose with available immunogenicity results. According-to-protocol (ATP) cohort for immunogenicity: all evaluable subjects (i.e., those meeting all eligibility criteria, with no elimination criteria during the relevant analysis interval), who received two vaccine doses and for whom assay results were available at Day 42.

The ATP cohort for immunogenicity at Day 63 included 541 subjects. The reasons for removal of subjects from the immunogenicity analyses are shown in Figure 1.

Recruitment began October 13, 2009 and the last study visit occurred May 25, 2010 (Day 0 to Day 182).

Baseline data is seen in Table 1.

**Table 1:** Summary of demographic characteristics (ATP cohort for immunogenicity)

**Characteristics**	**parameters or categories**	**A**		**B**		**C**		**D**		**E**		**F**		**Total**	
		**N = 92**		**N = 86**		**N = 92**		**N = 89**		**N = 91**		**N = 91**		**N = 541**	
		**Value or n**	**%**	**Value or n**	**%**	**Value or n**	**%**	**Value or n**	**%**	**Value or n**	**%**	**Value or n**	**%**	**Value or n**	**%**
Age (years)	Mean	29.6	-	28.8	-	28.0	-	29.3	-	29.6	-	28.5	-	29.0	-
	SD	6.30	-	6.46	-	6.41	-	6.21	-	6.45	-	6.19	-	6.34	-
	Median	29.5	-	28.0	-	27.0	-	29.0	-	29.0	-	27.0	-	28.0	-
	Minimum	19	-	19	-	19	-	19	-	19	-	19	-	19	-
	Maximum	40	-	40	-	40	-	40	-	40	-	40	-	40	-
Gender	Female	47	51.1	49	57.0	50	54.3	43	48.3	57	62.6	55	60.4	301	55.6
	Male	45	48.9	37	43.0	42	45.7	46	51.7	34	37.4	36	39.6	240	44.4
Ethnicity	American hispanic or latino	11	12.0	9	10.5	7	7.6	5	5.6	4	4.4	11	12.1	47	8.7
	Not american hispanic or latino	81	88.0	77	89.5	85	92.4	84	94.4	87	95.6	80	87.9	494	91.3
Geographic Ancestry	African heritage/african american	14	15.2	11	12.8	14	15.2	13	14.6	11	12.1	15	16.5	78	14.4
	American indian or alaskan native	1	1.1	0	0.0	0	0.0	0	0.0	0	0.0	0	0.0	1	0.2
	Asian - central/south asian heritage	0	0.0	0	0.0	0	0.0	0	0.0	0	0.0	1	1.1	1	0.2
	Asian - east asian heritage	0	0.0	1	1.2	1	1.1	1	1.1	0	0.0	1	1.1	4	0.7
	Asian - japanese heritage	0	0.0	0	0.0	0	0.0	0	0.0	0	0.0	0	0.0	0	0.0
	Asian - south east asian heritage	0	0.0	1	1.2	0	0.0	0	0.0	0	0.0	2	2.2	3	0.6
	Native hawaiian or other pacific islander	0	0.0	0	0.0	3	3.3	0	0.0	0	0.0	0	0.0	3	0.6
	White - arabic/north african heritage	1	1.1	0	0.0	0	0.0	0	0.0	0	0.0	0	0.0	1	0.2
	White - caucasian/european heritage	74	80.4	72	83.7	73	79.3	75	84.3	78	85.7	70	76.9	442	81.7
	Other	2	2.2	1	1.2	1	1.1	0	0.0	2	2.2	2	2.2	8	1.5

A = TIV+Pl d0, 15 mcg d21,42.

B = TIV+Pl d0, 3.75 mcg Adj d21,42.

C = 15 mcg + TIV d0, 15 mcg d21, Pl d42.

D = 3.75 mcg Adj + TIV d0, 3.75 mcg Adj d21, Pl d42.

E = 15 mcg + Pl d0, 15 mcg d21, TIV d42.

F = 3.75 mcg Adj + Pl d0, 3.75 mcg Adj d21, TIV d42.

N = total number of subjects.

n/% = number/percentage of subjects in a given category.

Value = value of the considered parameter.

*SD* = standard deviation.

### Outcomes

#### Immunogenicity

##### Co-administration of TIV with 15 μg unadjuvanted or AS03-adjuvanted A(H1N1)pmd09 vaccines: effect on immune response to A(H1N1)pmd09 HA antigen

Co-administration of TIV with the non-adjuvanted 15 μg HA A(H1N1)pmd09 vaccine did not influence the immune responses to A(H1N1)pmd09 HA antigen. Non-inferiority was demonstrated between participants receiving two 15 μg doses of non-adjuvanted A(H1N1)pmd09 vaccine 21 days apart with the first dose co-administered with TIV (Group C), and those receiving two 15 μg doses of non-adjuvanted H1N1 2009 vaccine 21 days apart without TIV (Group E). The lower bound of 97.5% CI for group GMT ratio at Day 42 was >0.5 (Table 2). Although non-inferiority was demonstrated according to protocol-specified criteria, the point estimate of GMT was 30% lower when TIV was co-administered with non-adjuvanted pandemic vaccine (Group C), compared to when A(H1N1)pmd09 vaccine was administered alone.

**Table 2:** Non-inferiority outcomes of Haemagglutination Inhibition (HI) antibody geometric mean titres (GMT) ratio (ATP cohort for immunogenicity)

**Ratio order**	**Strain**	**Adjusted GMT**^**a**^**ratio**	**Non-inferiority criteria**
**TIV**^b^**co-administration**		**Value [97.5% CI**^**c**^**]**	
Group D Day 42/Group F Day 42	A/California/7/2009	0.62 **[0.48**-0.81]	If the lower bound of 95% CI for GMT ratio between Group D Day 42; Group F Day 42 was >0.5
Group C Day 42/Group E Day 42	A/California/7/2009	0.66 [0.51-0.85]	If the lower bound of 95% CI for GMT ratio between Group C Day 42; Group E Day 42 was >0.5
***Sequential administration***		**Value [95% CI]**	
Group B Day 63/Group F Day 42	A/California/7/2009	0.68 [0.54-0.86]	If the lower bound of 95% CI for GMT ratio between Group B Day 63; Group F Day 42 was >0.5
(TIV prior to adjuvanted H1N1 vaccine)			
Group A Day 63/Group E Day 42	A/California/7/2009	0.60 [**0.48**-0.76]	If the lower bound of 95% CI for GMT ratio between Group A Day 63; Group E Day 42 was >0.5
(TIV prior to unadjuvanted H1N1 vaccine)			
Group B Day 63/Group A Day 63	A/California/7/2009	2.69 [2.14-3.39]	If the lower bound of 95% CI for GMT ratio between Group B Day 63; Group A Day 63 was >0.5; Superiority criteria: Lower bound >1.0
(adjuvanted H1N1/unadjuvanted H1N1)			
***Immune response to seasonal influenza strains***		**Value [95% CI]**	
Group D Day 21/Group A+B Day 21	A/Brisbane/59/2007	0.91 [0.70-1.17]	If the lower bound of 95% CI for GMT ratio between Group D Day 21; Group A+B Day 21 was >0.5
(co-administration, adjuvanted)	A/Uruguay/716/2007	1.15 [0.81-1.63]	
	B/Brisbane/60/2008	0.92 [0.74-1.15]	
Group C Day 21/Group A+B Day 21	A/Brisbane/59/2007	1.23 [0.96-1.58]	If the lower bound of 95% CI for GMT ratio between Group C Day 21; Group A+B Day 21 was >0.5
(co-administration, unadjuvanted)	A/Uruguay/716/2007	1.15 [0.81-1.63]	
	B/Brisbane/60/2008	0.74 [0.60-0.92]	
Group F Day 63/Group A+B Day 21	A/Brisbane/59/2007	0.96 [0.75-1.24]	If the lower bound of 95% CI for GMT ratio between Group F Day 63; Group A+B Day 21 was >0.5
(Sequential adjuvanted)	A/Uruguay/716/2007	1.20 [0.85-1.69]	
	B/Brisbane/60/2008	1.00 [0.81-1.24]	
Group E Day 63/Group A+B Day 21	A/Brisbane/59/2007	0.84 [0.65-1.08]	If the lower bound of 95% CI for GMT ratio between Group E Day 63; Group A+B Day 21 was >0.5
(Sequential unadjuvanted)			
	A/Uruguay/716/2007	1.24 [0.88-1.76]	
	B/Brisbane/60/2008	0.94 [0.76-1.17]	

^a^*GMT*: Geometric Mean Titre.

^b^*TIV*: Trivalent inactivated influenza vaccine.

^c^*CI*: Confidence Interval.

BOLD : values of adjusted GMT ratio that did not met the pre-specified criteria.

Group definitions:

Group A: TIV+Placebo (Day 0); 15 μg (Day 21); 15 μg (Day 42).

Group B: TIV+Placebo (Day 0); 3.75 μg/AS03 (Day 21); 3.75 μg/AS03 (Day 42).

Group C: 15 μg+TIV (Day 0); 15 μg (Day 21); Placebo (Day 42).

Group D: 3.75 μg/AS03+TIV (Day 0); 3.75 μg/AS03 (Day 21); Placebo (Day 42).

Group E: 15 μg+Placebo (Day 0); 15 μg (Day 21); TIV (Day 42).

Group F: 3.75 μg/AS03+Placebo (Day 0); 3.75 μg/AS03 (Day 21); TIV (Day 42).

Non-inferiority was not established between sub-jects receiving two doses of AS03-adjuvanted 3.75 μg HA A(H1N1)pmd09 vaccine with the first dose co-administered with TIV (Group D), and subjects receiving two doses of AS03-adjuvanted 3.75 μg HA A(H1N1)pmd09 vaccine without TIV (Group F). The lower bound of 97.5% CI for group GMT ratio at Day 42 was marginally <0.5 (Table 2). The A/California/7/2009 GMTs at Day 42 were about 37% lower when TIV was co-administered with AS03-adjuvanted A(H1N1)pmd09 vaccine (Group D).

##### Previous TIV vaccination followed by 15 μg unadjuvanted or AS03-adjuvanted A(H1N1)pmd09 vaccines: effect on immune response to A(H1N1)pmd09 HA antigen

Subjects who had TIV followed by two doses of non-adjuvanted A(H1N1)pmd09 vaccine (Group A) were not non-inferior compared to those who had two doses of non-adjuvanted A(H1N1)pmd09 vaccine with TIV at Day 42 (Group E), (Table 2). A(H1N1)pmd09 specific HI GMTs were lower when TIV was administered prior to 15 μg A(H1N1)pmd09 pandemic vaccine than when H1N1 2009 vaccine was administered alone (33% lower for Group A on Day 42 vs Group E on Day 21 and 34% lower for Group A on Day 63 vs Group E on Day 42 Table 3).

**Table 3:** Haemagglutination inhibition antibodies against vaccine homologous A/California/7/2009 strain [CHMP/CBER criteria] (According To Protocol cohort for immunogenicity)

**Immune response**	**Time point**	**Group A**	**Group B**	**Group C**	**Group D**	**Group E**	**Group F**
		**Day 0: TIV**^**a**^**+Placebo**	**Day 0: TIV +Placebo**	**Day 0: 15 μg +TIV**	**Day 0: 3.75 μg/ AS03+TIV**	**Day 0: 15 μg +Placebo**	**Day 0: .75 μg/ AS03+Placebo**
		**Day 21: 15 μg**	**Day 21: 3.75 μg/AS03**	**Day 21: 15 μg**	**Day 21: 3.75 μg/AS03**	**Day 21: 15 μg**	**Day 21: 3.75 μg/AS03**
		**Day 42: 15 μg**	**Day 42: 3.75 μg/AS03**	**Day 42: Placebo**	**Day 42: Placebo**	**Day 42: TIV**	**Day 42: TIV**
		**Value or % [95% CI**^**b**^**]**					
	Day 21	N^a^=92	N=86	N=92	N=88	N=91	N=91
	Day 42	N=92	N=86	N=92	N=88	N=91	N=91
	Day 63	N=92	N=86	N=92	N=88	N=91	N=91
	Day 182	N=92	N=89	N=85	N=88	N=89	N=91
**Seroconversion rate**	Day 21	**20.7%**	**23.3%**	87.0%	95.5%	93.4%	97.8%
[CBER^c^: LL^e^ of 95% CI >40%]							
		**[12.9-30.4%]**	**[14.8-33.6%]**	[78.3-93.1%]	[88.8-98.7%]	[86.2-97.5%]	[92.3-99.7%]
[CHMP^d^: point estimate >40%]	Day 42	85.9%	96.5%	91.3%	96.6%	96.7%	98.9%
		[77.0-92.3%]	[90.1-99.3%]	[83.6-96.2%]	[90.4-99.3%]	[90.7-99.3%]	[94.0-100%]
	Day 63	85.9%	97.7%	91.3%	95.5%	94.5%	98.9%
		[77.0-92.3%]	[91.9-99.7%]	[83.6-96.2%]	[88.8-98.7%]	[87.6-98.2%]	[94.0-100%]
	Day 182	76.1%	87.6%	78.8%	89.8%	87.6%	92.3%
		[66.1-84.4%]	[79.0-93.7%]	[68.6-86.9%]	[81.5-95.2%]	[79.0-93.7%]	[84.8-96.9%]
**Seroprotection rate**	Day 0	25.0%	10.5%	19.6%	18.2%	13.2%	13.2%
[CBER: LL of 95% CI >70%]							
		[16.6-35.1%]	[4.9-18.9%]	[12.0-29.1%]	[10.8-27.8%]	[7.0-21.9%]	[7.0-21.9%]
[CHMP: point estimate >70%]	Day 21	**48.9%**	**39.5%**	96.7%	98.9%	97.8%	100%
		**[38.3-59.6%]**	**[29.2-50.7%]**	[90.8-99.3%]	[93.9-100%]	[92.3 -99.7%]	[96.0-100%]
	Day 42	97.8%	100%	100%	100%	100%	100%
		[92.4-99.7%]	[95.8-100%]	[96.1-100%]	[95.9-100%]	[96.0-100%]	[96.0-100%]
	Day 63	97.8%	100%	100%	100%	98.9%	100%
		[92.4-99.7%]	[95.8-100%]	[96.1-100%]	[95.9-100%]	[94.0-100%]	[96.0-100%]
	Day 182	90.2%	97.8%	92.9%	100%	94.4%	97.8%
		[82.2-95.4%]	[92.1-99.7%]	[85.3-97.4%]	[95.9-100%]	[87.4-98.2%]	[92.3-99.7%]
**Geometric mean fold rise**	Day 21	**2.1**	**2.4**	21.3	33.7	38.4	65.3
		**[1.8-2.6]**	**[1.9-3.0]**	[16.4-27.7]	[26.0-43.6]	[29.3-50.5]	[51.1-83.5]
[CHMP: point estimate >2.5]							
	Day 42	16.3	36.5	20.9	54.9	41.5	92.4
		[12.6-21.1]	[28.9-46.0]	[16.3-26.7]	[42.1-71.7]	[32.4-53.2]	[73.3-116.3]
	Day 63	17.4	59.4	18.8	39.8	37.0	69.7
		[13.4-22.6]	[46.6-75.7]	[14.7-24.1]	[31.2-50.7]	[28.6-47.9]	[55.1-88.1]
	Day 182	9.5	17.7	11.1	15.1	21.9	32.5
		[7.3-12.4]	[14.3-21.8]	[8.6-14.4]	[11.9-19.1]	[16.4-29.4]	[26.0-40.7]
**Geometric mean titres**	Day 0	14.6	10.9	13.0	10.7	9.3	10.1
		[11.2-19.0]	[8.7-13.6]	[10.2-16.5]	[8.2-14.0]	[7.7-11.4]	[8.2-12.5]
	Day 21	31.1	26.3	277.3	361.0	358.7	659.8
		[24.0-40.3]	[19.9-34.8]	[222.4-345.7]	[301.5-432.2]	[285.0 -451.4]	[546.0-797.4]
	Day 42	238.6	396.2	271.2	589.8	387.1	933.1
		[191.8-296.7]	[329.2-476.8]	[224.6-327.4]	[517.8-671.8]	[314.4-476.7]	[815.6-1067.6]
	Day 63	254.2	645.2	245.0	426.9	345.3	704.0
		[210.1-307.7]	[557.6-746.6]	[200.7-299.1]	[373.0-488.5]	[278.6-428.0]	[602.1-823.1]
	Day 182	136.6	206.9	151.7	171.7	218.5	323.6
		[108.6-171.9]	[171.3-249.9]	[119.1-193.2]	[143.2-205.8]	[169.8-281.1]	[268.8-389.5]

^a^*N* = Number of subjects with available results.

^b^*CI* = Confidence Interval.

^c^*CBER* = Center for Biologics Evaluation & Research.

^d^*CHMP*: Committee for Medicinal Products for Human Use.

^e^*LL* = Lower limit.

BOLD : values of SPR and Geometric mean fold rise that did not meet the pre-specified criteria.

Subjects immunized first with TIV followed by two doses of AS03-adjuvanted A(H1N1)pmd09 vaccine (Group B), had non-inferior immune responses to the pandemic strain compared to those subjects receiving two doses of AS03-adjuvanted A(H1N1)pmd09 vaccine without previous TIV receipt (Group F). However, A(H1N1)pmd09 specific HI GMTs were lower when TIV was administered prior to adjuvanted A(H1N1)pmd09 pandemic vaccine than when adjuvanted A(H1N1)pmd09 vaccine was administered alone (40% lower for Group B on Day 42 vs Group F on Day 21and 31% lower for Group B on Day 63 vs Group F on Day 42 Table 3).

Subjects who had TIV followed by two doses of AS03-adjuvanted 3.75 μg HA A(H1N1)pmd09 vaccine (Group B) had superior A(H1N1)pmd09 specific HI GMTs compared to those who had TIV followed by non-adjuvanted pandemic vaccine (Group A). The lower bound of 95% CI for group GMT ratio was >1.0 (Table 2).

Although not a planned comparison, it is noted that A(H1N1)pmd09 specific HI GMTs were superior when TIV was given with adjuvanted vaccine (Group C) compared to unadjuvanted vaccine (Group D) (Table 3).

##### Co-administration of TIV with A(H1N1)pmd09 vaccines: effect on TIV strain immunogenicity

The immune responses to the three TIV strains did not change when TIV was co-administered with the A(H1N1)pmd09 vaccine whether AS03-adjuvanted (Group D) or non-adjuvanted (Group C) and non-inferiority was established in all planned comparisons compared to subjects administered a single dose of TIV (Groups A and B pooled) (Table 2).

##### A(H1N1)pmd09 vaccine followed by TIV: effect on immune response to TIV strains

Previous vaccination with A(H1N1)pmd09 vaccine did not influence the immune responses to the TIV strains. Non-inferiority was established between subjects who received two doses of AS03-adjuvanted or non-adjuvanted A(H1N1)pmd09 vaccine followed by TIV (Groups E or F), and subjects receiving a single dose of TIV (Groups A and B pooled)(Table 2).

##### CHMP and CBER criteria

All six groups met the CHMP and CBER immunogenicity guidance criteria for the A(H1N1)pmd09 HA antigen, 21 days after the first dose of the AS03-adjuvanted or non-adjuvanted formulations (Table 2 and additional file 1). Following the second vaccine dose, HI titres increased further.

For the TIV strains, the CHMP and CBER criteria were met in all groups, 21 days after TIV administration (Day 42 or Day 63) except in Group F against A/Brisbane/59/2007 (SCR: 36.3% at Day 63 using Day 42 as baseline, but 83.5% and surpassing criteria at Day 63 using Day 0 as baseline) (Table 4).

**Table 4:** Haemagglutination inhibition antibody immune responses against TIV strains (According To Protocol cohort for immunogenicity)

**Strain**	**Time point**	**Group**	**N**^**a**^	**Seroconversion rate**	**Seroprotection rate**	**Geometric mean fold rise >2.5**	**Geometric mean titre**
				**Value or % [95% CI**^**a**^**]**			
A/Brisbane/59/2007	Day 21	A	92	77.2% [67.2-85.3%]	93.5% [86.3-97.6%]	8.4 [6.5-10.8]	145.1 [115.9-181.6]
		B	86	86.0% [76.9-92.6%]	97.7% [91.9-99.7%]	12.9 [10.0-16.6]	215.7 [168.2-276.5]
		C	92	82.6% [73.3-89.7%]	96.7% [90.8-99.3%]	12.0 [9.4-15.4]	226.3 [181.4-282.2]
		D	88	79.5% [69.6-87.4%]	92.1% [84.5-96.8%]	9.6 [7.7-12.1]	158.7 [127.9-197.1]
	Day 42	E	91	18.7% [11.3-28.2%]	51.6% [40.9-62.3%]	2.3 [1.9-2.6]	32.9 [27.3-39.7]
		F	91	30.8% [21.5-41.3]	68.1% [57.5-77.5%]	3.2 [2.7-3.8]	53.2 [43.5-65.1]
	Day 63	A	92	68.5% [58.0-77.8%]	91.3% [83.6-96.2%]	6.9 [5.4-8.9]	120.2 [96.5-149.7]
		B	86	88.4% [79.7-94.3%]	100% [95.8-100%]	11.2 [9.1-13.7]	186.5 [150.4-231.4]
		C	92	75.0% [64.9-83.4%]	92.4% [84.9-96.9%]	8.4 [6.6-10.6]	157.0 [126.0-195.8]
		D	88	72.7% [62.2-81.7%]	88.8%[80.3-94.5%]	7.6 [6.0-9.5]	124.3 [99.0-156.1]
		E	91	78.0% [68.1-86.0%]	89.0% [80.7-94.6%]	9.4 [7.6-11.7]	137.4 [109.0-173.1]
		F	91	83.5% [74.3-90.5%]	95.6% [89.1-98.8%]	10.1 [8.1-12.6]	166.9 [132.5-210.3]
	Day 182	A	92	48.9% [38.3-59.6%]	68.5% [58.0-77.8%]	4.0 [3.1-5.3]	68.5 [53.6-87.4]
		B	89	64.0% [53.2-73.9%]	83.1% [73.7-90.2%]	5.2 [4.2-6.6]	89.2 [71.0-112.0]
		C	85	52.9% [41.8-63.9%]	77.6% [67.3-86.0%]	4.7 [3.7-6.0]	87.4 [68.5-111.5]
		D	89	43.2% [32.7-54.2%]	68.5% [57.8-78.0%]	3.7 [2.9-4.8]	64.6 [51.4-81.1]
		E	89	51.7% [40.8-62.4%]	75.3% [65.0-83.8%]	4.4 [3.5-5.4]	64.6 [51.4-81.1]
		F	91	59.3% [48.5-69.5%]	78.0% [68.1-86.0%]	5.0 [4.0-6.3]	83.7 [66.0-106.2]
A/Uruguay/716/2007	Day 21	A	92	78.3% [68.4-86.2%]	84.8% [75.8-91.4%]	17.4 [13.0-23.3]	175.7 [129.6-238.2]
		B	86	84.9% [75.5-91.7%]	89.5% [81.1-95.1%]	15.5 [11.8-20.4]	183.5 [135.2-248.9]
		C	92	78.3% [68.4-86.2%]	83.7% [74.5-90.6%]	20.6 [14.8-28.6]	181.7 [128.1-257.9]
		D	88	86.4% [77.4-92.8%]	88.8% [80.3-94.5%]	19.3 [14.2-26.4]	191.4 [138.4-264.7]
	Day 42	E	91	3.3% [0.7-9.3%]	19.8% [12.2-29.4%]	1.2 [1.1-1.4]	12.5 [10.0-15.6]
		F	91	6.6% [2.5-13.8%]	30.8% [21.5-41.3%]	1.7 [1.5-1.9]	19.1 [15.6-23.4]
	Day 63	A	92	77.2% [67.2-85.3%]	84.8% [75.8-91.4%]	13.3 [10.0-17.6]	134.4 [100.4-179.9]
		B	86	80.2% [70.2-88.0%]	87.2% [78.3-93.4%]	11.7 [9.1-15.0]	138.4 [104.8-182.7]
		C	92	75.0% [64.9-83.4%]	79.3% [69.6-87.1%]	15.0 [11.2-19.9]	132.0 [94.9-183.7]
		D	88	78.4% [68.4-86.5%]	84.3% [75.0-91.1%]	14.6 [10.9-19.6]	145.6 [107.7-196.8]
		E	91	82.4% [73.0-89.6%]	89.0% [80.7-94.6%]	20.8 [15.5-28.0	216.1 [160.0-292.0]
		F	91	85.7% [76.8-92.2%]	91.2% [83.4-96.1%]	19.3 [14.6-25.5]	221.9 [167.2-294.6]
	Day 182	A	92	63.0% [52.3-72.9%]	71.7% [61.4-80.6%]	9.0 [6.7-11.9]	89.5 [65.8-121.8]
		B	89	62.9% [52.0-72.9%]	71.9% [61.4-80.9%]	7.2 [5.6-9.3]	87.7 [64.0-120.3]
		C	85	63.5% [52.4-73.7%]	70.6% [59.7-80.0%]	9.7 [7.2-13.1]	87.1 [61.4-123.5]
		D	89	63.6% [52.7-73.6%	71.9% [61.4-80.9%]	8.5 [6.2-11.7]	87.8 [63.4-121.4]
		E	89	67.4% [56.7-77.0%]	76.45 [66.2-84.85]	9.9 [7.3-13.5]	110.0 [79.3-152.6]
		F	91	67.0% [56.4-76.5%]	76.9% [66.9-85.1%]	10.6 [7.9-14.2]	127.2 [92.3-175.5]
B/Brisbane/60/2008	Day 21	A	92	82.6% [73.3-89.7%]	98.9% [94.1-100%]	12.6 [9.8-16.2]	662.1 [545.4-803.8]
		B	86	88.4% [79.7-94.3%]	98.8% [93.7-100%]	13.9 [11.1-17.5]	658.3 [540.2-802.0]
		C	92	80.4% [70.9-88.0%]	97.8% [92.4-99.7%]	10.5 [8.3-13.5]	478.8 [389.5-588.6]
		D	88	88.6% [80.1-94.4%]	100% [95.9-100%]	14.3 [11.0-18.7]	576.1 [479.8-691.8]
	Day 42	E	91	6.6% [2.5-13.8%]	82.4% [73.0-89.65]	1.3 [1.1-1.5]	74.7 [61.4-90.9]
		F	91	26.4% [17.7-36.7%]	97.8% [92.3-99.7%]	2.4 [2.0-2.8]	139.5 [120.0-162.2]
	Day 63	A	92	79.3% [69.6-87.1%]	98.9% [94.1-100%]	9.6 [7.5-12.4]	506.7 [422.3-608.0]
		B	86	84.9% [75.5-91.7%]	100% [95.8-100%]	10.0 [8.1-12.2]	471.2 [396.3-560.2]
		C	92	72.8% [62.6-81.6%]	98.9% [94.1-100%]	7.9 [6.3-9.9]	359.6 [299.4-431.9]
		D	88	83.0% [73.4-90.1%]	100% [95.9-100%]	10.8 [8.4-13.7]	433.5 [369.5-508.7]
		E	91	85.7% [76.8-92.2%]	100% [96.0-100%]	11.4 [9.1-14.2]	644.9 [534.5-778.2]
		F	91	83.5% [74.3-90.5%]	100% [96.0-100%]	11.9 [9.4-15.0]	688.1 [593.0-798.6]
	Day 182	A	92	73.9% [63.7- 82.5%]	97.8% [92.4-99.7%]	6.7 [5.2-8.6]	339.9 [276.7-417.5]
		B	89	60.7% [49.7-70.9%]	98.9% [93.9-100%]	5.7 [4.5-7.1]	273.8 [228.7-327.9]
		C	85	56.5% [45.3-67.2%]	92.9% [85.3-97.4%]	5.5 [4.3-6.9]	245.4 [197.8-304.3]
		D	89	72.7% [62.2-81.7%]	100% [95.9-100%]	6.8 [5.3-8.6]	292.6 [247.0-346.7]
		E	89	75.3% [65.0-83.8%]	98.9% [93.9-100%]	6.9 [5.4-8.7]	369.6 [300.0-455.3]
		F	91	68.1% [57.5-77.5%]	100% [96.0-100%]	6.4 [5.0-8.1]	378.4 [323.4-442.8]

^a^*N* = Number of subjects with available results.

^b^*LL* = Lower limit.

^c^*CI* = Confidence Interval.

Six months after the first vaccine dose (Day 182), the immune responses to A(H1N1)pmd09 HA antigen in all groups continued to meet CHMP and CBER criteria.

#### Safety and reactogenicity

**Figure 2:**
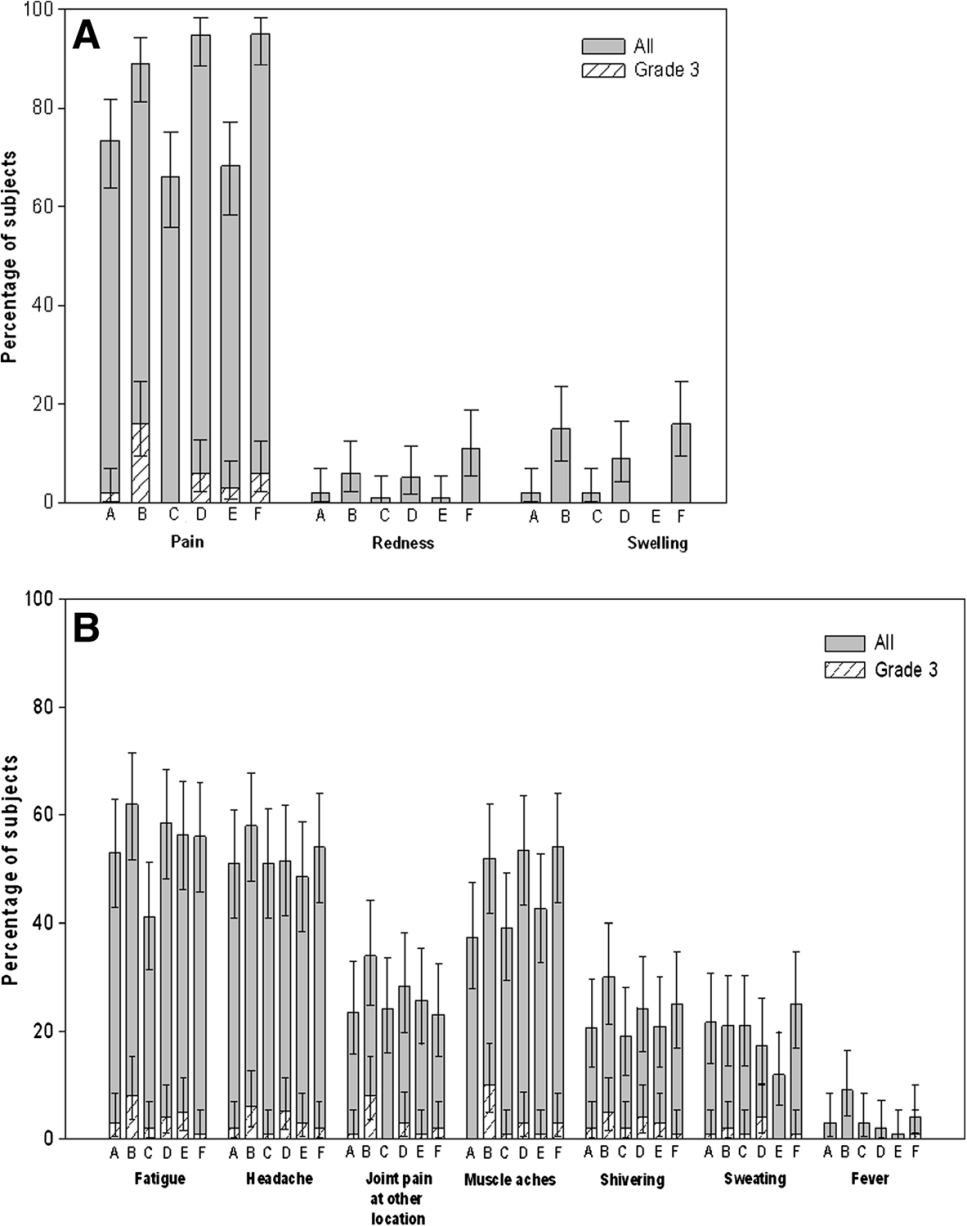
**Incidence and 95%CI of solicited local and general symptoms recorded during the 7-day post-vaccination follow-up period (Total vaccinated cohort).** Group definitions: Group A: TIV+Placebo (Day 0); 15 μg (Day 21); 15 μg (Day 42); Group B: TIV+Placebo (Day 0); 3.75 μg/AS03 (Day 21); 3.75 μg/AS03 (Day 42); Group C: 15 μg+TIV (Day 0); 15 μg (Day 21); Placebo (Day 42); Group D: 3.75 μg/AS03+TIV (Day 0); 3.75 μg/AS03 (Day 21); Placebo (Day 42); Group E: 15 μg+Placebo (Day 0); 15 μg (Day 21); TIV (Day 42); Group F: 3.75 μg/AS03+Placebo (Day 0); 3.75 μg/AS03 (Day 21); TIV (Day 42).

Solicited local symptoms were more common in recipients of AS03-adjuvanted vaccine compared to those who received the non-adjuvanted A(H1N1)pmd09 vaccine (86.5−95.0% v. 64.7−73.5%, subjects reported at least one local symptom respectively). Injection site pain was the most frequently reported solicited local symptom across all groups (Groups B, D, F: 89.0%, 94.9%, and 95.0%, respectively); injection site pain in recipients of non-adjuvanted A(H1N1)pmd09 vaccine ranged from 66 to 73.5%. Grade 3 injection site pain was reported by ≤6.1% of subjects across the groups, except Group B (16.0% reported Grade 3 pain) (Figure 2A).

The overall incidence of solicited general symptoms was similar in AS03-adjuvanted or non-adjuvanted A(H1N1)pmd09 vaccine groups (76.9−85.3% v. 77.2−78.4%, respectively). Fatigue and headache were the most frequently reported solicited general symptoms across all groups (41.0−58.6% and 48.5−58.0%, subjects reported at least one general symptom respectively); Grade 3 fatigue and headache were reported in ≤8.0% of subjects (Figure 2B).

Unsolicited adverse events were similar between groups up to Day 84 (55.9−66.7%). Nausea (Group A: 4.9%), cough (Group C: 2.9%; Group E: 3.0%), oropharyngeal pain (Group B: 6.7%; Group C: 2.9%; Group D: 7.0%; Group F: 4.9%), rhinorrhoea (Group C: 2.9%), nasal congestion (Group E: 3.0%) were the most frequently recorded unsolicited adverse events considered to be causally related to vaccination; <5.0% of subjects reported unsolicited adverse events of Grade 3 intensity that were considered to be causally related to vaccination by local investigators; 23.8−37.3% of subjects reported unsolicited adverse events that prompted a medical visit.

Seven serious adverse events including one potential immune-mediated-disease (pIMD) were recorded for six subjects to the Day 182 follow-up time point. One of the SAEs (subject with elevated alanine transaminase considered by the investigator to be of Grade 3 intensity and required medical attention) was considered by the investigator to be possibly vaccination-related. The single pIMD (facial palsy of Grade 2 intensity) was considered by the investigator to be unrelated to the study vaccines. All SAEs including the pIMD were resolved by Day 182. There were no clinically relevant trends in laboratory test results and no evidence of differential hepatic or renal toxicities associated with receipt of the adjuvant.

## Discussion

The logistics of delivering a mass immunization program are considerable, and concurrent administration of vaccines would be efficient for providers and more appealing to vaccinees than a requirement to make multiple visits to a healthcare provider. During the influenza A(H1N1)pmd09 pandemic, considerable variability in administration of TIV and pandemic vaccines occurred, with some authorities recommending co-administration, and others recommending that TIV be given a month or more after the pandemic vaccine. Of course, concurrent administration of vaccines is desirable only when immunogenicity is not adversely effected, and safety is similar or improved.

Although regulatory criteria for A(H1N1)pmd09 strain immunogenicity were met in all vaccine groups at 21 days after the first H1N1 dose and up to six months later, the HI antibody response against the pandemic strain following co-administration or sequential administration of TIV and A(H1N1)pmd09 vaccines was decreased compared to when A(H1N1)pmd09 vaccine was administered alone. Co-administration of TIV with the first dose of pandemic vaccine, or TIV administration 21 days prior to pandemic vaccination, appeared to negatively influence the immune response to the non-adjuvanted 15 μg HA A(H1N1)pmd09 vaccine and the AS03-adjuvanted 3.75 μg HA A(H1N1)pmd09 vaccine to a similar degree (range of GMT decrement 30.6% to 46.9%). This effect was similar at Day 63 and Day 182. This finding contrasts with data from studies of concurrent TIV and monovalent A(H1N1)pmd09 vaccination which reported that sequential and simultaneous administration of A(H1N1)pmd09 influenza vaccine and seasonal influenza vaccine did not influence the immune responses to either vaccine antigens [3,5-7] . In contrast to our study, participants in these cited studies could have received TIV in previous seasons compared to our population of TIV-naive subjects. As well an older age range of adults was included in other studies compared to our population of 18 to 40 year olds, and different study designs were used.

In a study in elderly adults (>64 years) using a similar AS03-adjuvanted dose-sparing vaccine, evidence of modest influence was also observed, though again with strong immune responses against the vaccine homologous A(H1N1)pmd09 strain when TIV was co-administered or sequentially administered with A(H1N1)pmd09 vaccine (GMTs: 227.5−309.8; SCRs: 64.2−97.6%) [5]. In a study of adults including persons over 60 years, co-administration of whole virion inactivated TIV and 6 μg HA inactivated whole virion H1N1 2009 vaccine adjuvanted with aluminium phosphate gel elicited strong immune responses against both H1N1 strains and seasonal influenza strains (GMFR: ~8.0 and 2.7−3.8; SCRs: ~81.0% and 40.3−70.7%, respectively) [8].

Interestingly, TIV vaccination prior to pandemic vaccine in these TIV-naïve study participants increased, moderately, A(H1N1)pmd09 specific HI titres in subjects on Day 21 even before they received adjuvanted and non-adjuvanted pandemic vaccines. Other studies also suggest that prior TIV vaccination is associated with increased pandemic specific antibody responses [9,10]. The mechanism of this phenomenon is not known, but could be explained by lack of specificity of HI antibody testing or cross-reactive antibody responses despite lack of epitope similarity. Since up to 25% of subjects across groups in the present study were seroprotected at baseline, likely due to natural infection during the first wave of the pandemic, many of these vaccine-naive participants could have had A(H1N1)pmd09 infection without it being physician-diagnosed.

Immunogenicity to the TIV strains was unaffected by co- or sequential administration with the AS03-adjuvanted and non-adjuvanted H1N1 2009 vaccines; the CHMP and CBER criteria were met in all groups, 21 days after TIV administration (Day 42 or Day 63) and up to at least six months following the first TIV dose, except in Group F against A/Brisbane/59/2007.

As has been previously demonstrated, use of an oil-in-water adjuvant was associated with more robust immune pandemic influenza A(H1N1)pmd09 responses at all time points compared to use of non-adjuvanted 15 μg HA A(H1N1)pmd09 vaccine (to be noted: 11 μg instead of 15 μg HA was delivered). This observation has been documented in all age groups tested and for influenza antigen doses of 3.75 μg and 1.9 μg HA [11,12].

The high frequency of injection site pain with the AS03-adjuvanted vaccine, is a consistent finding and in agreement with other reports [10-12]. Grade 3 injection site pain was uncommon. The incidence of general symptoms between adjuvanted and unadjuvanted vaccine recipients were comparable.

### Limitations

There are several limitations to our study. The sample included TIV-naïve subjects, that is, adults less than 40 years of age without a history of receiving TIV or the pandemic vaccine, in order to increase the likelihood that subjects had little or no prior exposure to influenza and reduce age-based immunogenicity-variation and allowing for a smaller sample size. About 10.5 to 25% of subjects were not naïve to the pandemic strain. Thus, these results may not be generalizable to older adults or those with prior influenza vaccine exposure. Also, the pandemic virus was circulating during the trial, and may have confounded vaccine immunogenicity. However this is not likely to have affected one or more of the vaccine groups disproportionately.

## Conclusions

This study shows that the co-administration of the A(H1N1)pmd09 vaccine with TIV did not alter the quantitative immune response to TIV strains but influenced immune response to A(H1N1)pmd09 strain. It is not known if clinical protection would be affected by this reduced immune response. Despite evidence of a lowered immune response, both formulations met the CHMP and CBER criteria for pandemic influenza vaccines, and continued to do so up to six months after Dose 1 of vaccine. HI antibody GMTs were higher at all time points following vaccination with the AS03-adjuvanted 3.75 μg A(H1N1)pmd09 vaccine compared to the non-adjuvanted 15 μg A(H1N1)pmd09 vaccine.

## Methods

### Trial design

This was a randomised, observer-blind, controlled clinical trial (Clinical trials registration NCT00985673) conducted at four sites in the United States and three in Canada (Table 5). Participants were randomised (allocation ratio 1:1:1:1:1:1) into six study groups (Groups A to F; Figure 1).

**Table 5:** Study sites

***Country***	***Province or State***	***Number of sites***	***Participants enrolled***
Canada	Quebec	2	225
	Nova Scotia	1	61
USA	Texas	2	195
	North Carolina	1	37
	Georgia	1	93
Total		7	611

#### Participants

Adults aged 19 to 40 years at the time of Dose 1 were eligible if they were in stable health with a satisfactory baseline medical assessment by history and physical examination, safety laboratory results were within protocol-specified limits and for women the pregnancy test was negative and there was agreement to continue adequate contraception for the study duration. Stable health was defined as the absence of a health event satisfying the definition of a serious adverse event (SAE), or a change in ongoing drug therapy due to therapeutic failure or symptoms of drug toxicity within one month prior to enrolment.

Exclusion criteria were a history of A(H1N1)pmd09 influenza vaccination or physician-confirmed infection, prior receipt at any time of a seasonal influenza vaccine, administration of any licensed vaccine within four weeks, or any investigational product within 30 days preceding Dose 1, immunodeficient condition or receipt of immunosuppressive drugs or of immunoglobulins/blood products, diagnosis of cancer, allergy to vaccine constituents, pregnancy, acute febrile illness, unstable psychiatric illness, disorder of coagulation or evolving neurologic disorder.

Written informed consent was obtained from all subjects prior to enrollment. The study was conducted in accordance with Good Clinical Practice and the Declaration of Helsinki. All study-related documents were approved by the appropriate Institutional Review Boards: the IWK Health Centre Research Ethics Board (Halifax, Nova Scotia, Canada), IRB Services (Aurora, Ontario) and Chesapeake Institutional Review Board (Austin, Texas).

### Interventions: study vaccines and immunization schedule

The A(H1N1)pmd09 pandemic influenza vaccine was a monovalent, inactivated, split-virion antigen without or with AS03 adjuvant system (*Arepanrix*™, GlaxoSmithKline Vaccines). The H1N1 viral seed for the vaccine was prepared from the reassortant virus NYMC X-179A (New York Medical College, New York) generated from the A(H1N1)pmd09 strain, as recommended by the World Health Organization (WHO) [4], and propagated in embryonated hen eggs. AS03_A_ is an Adjuvant System containing α-tocopherol and squalene in an oil-in-water emulsion (11.86 mg tocopherol). The AS03-adjuvanted formulations were prepared on the day of vaccine administration by an unblinded study nurse, by mixing the antigen suspension and adjuvant emulsion (1:1).

The antigen suspensions were manufactured to contain 15 μg/mL or 30 μg/mL haemagglutinin (HA) antigen; AS03-adjuvanted vaccine was prepared from the 15 μg/mL suspension (3.75 μg HA/0.25 mL+0.25 ml AS03: total vaccine dose=0.5 mL), while the non-adjuvanted formulation was prepared from the 30 μg/mL suspension (15 μg HA/0.5 mL). Approximately 11 months after release and following administration of Dose 1 (Day 0), routine antigen stability testing using the single radial immunodiffusion (SRID) assay indicated that the 30 μg/mL lot had less than targeted HA content. As a result, 11 μg instead of 15 μg HA was administered to subjects in Groups A, C and E. Antigen potency was re-assessed by SRID after completion of vaccination and remained stable at the same reduced level of HA content. In this paper this formulation will be referred by its intended dosage (15 μg). The HA antigen content of the 15 μg/mL HA lot remained stable and within the expected range.

The TIV was a split-virion vaccine (*FluLaval*™, GlaxoSmithKline Vaccines) formulated from the seasonal strains A/Brisbane/59/2007, A/Uruguay/716/2007 and B/Brisbane/60/2008. The antigen suspensions were manufactured to contain 15 μg HA of each strain per 0.5 mL doses.

Each 0.5 mL dose of A(H1N1)pmd09 vaccine and TIV contained 5 μg and 50 μg of thimerosal as preservative, respectively. All vaccines were administered intramuscularly in the deltoid of the arm. The first study vaccines were administered in each arm (Day 0), the second study vaccine in the dominant arm (Day 21), and the final vaccine (Day 42) in the non-dominant arm.

The monovalent pandemic vaccine without adjuvant, and the TIV were translucent to whitish suspensions. The AS03 is a white liquid, and when mixed with influenza antigen is a whitish emulsion.

### Outcomes

#### Immunogenicity assessments

Serum samples were collected before vaccination, 21 days after each vaccine dose and six months after the first vaccine dose (Days 0, 21, 42, 63 and 182) and tested in duplicate for haemagglutination inhibition (HI) titres using a validated assay [cut-off: ≥1:10] with chicken erythrocytes as previously described [13] at GSK laboratory (earlier timepoints tested together and D182 timepoint tested later).

The magnitude of immune responses was evaluated based on the immunogenicity criteria for pandemic influenza vaccines in adults as required by the Committee for Medicinal Products for Human Use (CHMP/EMA) [14]; point estimates for HI antibody seroconversion rate [SCR]: >40%, seroprotection rate [SPR]: >70% and geometric mean fold rise [GMFR]: >2.5) and the criteria for seasonal influenza vaccines in adults as required by Center for Biologics Evaluation and Research (CBER/USFDA; lower bound of 95% confidence interval [CI] for HI antibody for SCR: ≥40% and SPR: ≥70%) [15]. SCR was defined as the percentage of subjects with pre-vaccination titre <1:10 and post-vaccination titre ≥1:40, or pre-vaccination titre >1:10 and at least four-fold increase in post-vaccination titre, SPR as percentage of subjects with a post-vaccination titre ≥1:40 and GMFR as post-vaccination fold increase in geometric mean titres (GMTs).

#### Safety and reactogenicity assessments

Subjects used diary cards to record the occurrence and intensity of solicited local and general symptoms and unsolicited adverse events during the 7 and 21 day follow-up period after each vaccine dose, and until Day 84. Intensity of solicited symptoms was graded on a standard scale of [0–3]; Grade 1 symptoms defined as those that were noticeable but did not interfere with normal activities and Grade 3 symptoms defined as those that prevented normal activities (Grade 3 redness and swelling: diameter >100 mm; Grade 3 fever: temperature ≥39 °C [≥102.2 °F]). Serious adverse events and potential immune-mediated diseases (pIMDs: subset of adverse events that include both autoimmune diseases and other inflammatory and/or neurologic disorders which may or may not have an autoimmune etiology) occurring throughout the study period were also recorded. Clinical laboratory parameters were assessed at all seven visits up to Day 182.

### Sample size

A sample size objectives of 600 subjects was estimated to provide a power of 88.35% to evaluate each of the co-primary objectives. The unevaluable subject rate was estimated at ≤5%, the Log Standard Deviation for the GMT assumed to be 0.6, and the type 1 error of 0.025. The two co-primary objectives were evaluated in parallel, in terms of GMT ratio adjusted by pre-vaccination antibody titre. The study objective was considered met if one of the co-primary objectives was met. Hence, 97.5% confidence intervals (CIs) was used for the primary objective evaluation.

### Randomisation

Patients were randomized 1:1:1:1:1:1 to the six study groups. The randomization was performed by the sponsor using MATEX, a program develop for use in SAS (Cary, NC, USA) by GlaxoSmithKline (GSK) Vaccines, Belgium which incorporated a minimization algorithm. Study supplies were distributed to each study center by blocks containing all vaccine supplies.

Patients were enrolled by trained study personnel. Once eligibility was confirmed, specific unblinded study personnel were responsible for determining vaccine allocation as assigned by the internet based randomization system at each vaccination visit, vaccine preparation, and administration. The unblinded staff accessed the randomization system on the internet and provided the age and identification number of the participant. The randomization system assigned a treatment number which mapped to a vial number corresponding to supplies at that study site. Vaccine reconstitution by the unblinded nurse was done in a secure room, and then the individual dose carried to the participant’s room on a tray covered by an opaque cloth. The unblinded personnel had no other role in the study.

Neither the subjects or blinded study personnel evaluating the safety and immunogenicity endpoints were aware of vaccine assignment until the data analysis was completed.

### Statistical methods

The primary objectives of this study were to evaluate whether the immune response to A/California/07/2009 HA antigen in subjects who received a co-administration of TIV with the first of two doses of either the non-adjuvanted 15 μg HA vaccine or AS03-adjuvanted 3.75 μg HA vaccine (Groups C or D) was non-inferior compared to that in subjects who received two doses of these pandemic vaccines without TIV co-administration (Groups E or F). The co-primary objectives were evaluated by the group GMT ratio which was estimated via Analysis of covariance (ANCOVA) model on the logarithm transformed titres, with post-vaccine dose as a response (dependent variable) and baseline antibody level and vaccine group as the independent variable (included all the vaccine groups). The type-I error was adjusted for the parallel co-primary objectives. The non-inferiority criteria would be met if the lower bound of the 97.5% CI for GMR ratio at Day 42 between two groups (Groups D/F) OR (Groups C/E) was > 0.5.

The primary analyses of immunogenicity was performed on the According-To-Protocol (ATP) cohort, and of safety on the Total-Vaccinated-Cohort (TVC).

Secondary objectives included evaluation of the effect of TIV vaccination 21 days prior to pandemic vaccination on the immune response to the A/California/07/2009 HA antigen (Group A versus Group E and Group B versus Group F as described above) and evaluation of the effects of co-administration of pandemic vaccine and TIV (Groups C or D) or sequential administration (Groups E or F) on the immune response to each of the three TIV HA antigens as compared to no previous pandemic vaccine administration (Groups A and B).

Solicited local, general symptoms and unsolicited adverse events were summarized by vaccine group. No formal statistical comparisons were performed between groups for safety and reactogenicity (Table 6).

**Table 6:** Group names and vaccines received on Days 0, 21 and 42

**Group names**	**Vaccines received**		
	**Dose 1 (Day 0)**	**Dose 2 (Day 21)**	**Dose 3 (Day 42)**
Group A	TIV+Placebo	15 μg	15 μg
Group B	TIV+Placebo	3.75 μg/AS03	3.75 μg/AS03
Group C	15 μg+TIV	15 μg	Placebo
Group D	3.75 μg/AS03+TIV	3.75 μg/AS03	Placebo
Group E	15 μg+Placebo	15 μg	TIV
Group F	3.75 μg/AS03+Placebo	3.75 μg/AS03	TIV

## Abbreviations

ATP: According To Protocol; CBER: Center for Biologics Evaluation & Research; CHMP: Committee for Medicinal Products for Human Use; CI: Confidence Interval; HA: Haemagglutinin; HI: Haemagglutination Inhibition; SD: Standard Deviation; SRID: Single radial immunodiffusion; TIV: Trivalent Inactivated Vaccine; TVC: Total Vaccinated Cohort; WHO: World Health Organization.

## Competing interests

The study was funded by the US Department of Health and Human Services (HHS), Assistant Secretary of Preparedness and Response (ASPR), Biomedical Advanced Research and Development Authority (BARDA) and GlaxoSmithKline Biologicals SA. GlaxoSmithKline Biologicals SA, was involved in all stages of the study conduct and analysis (ClinicalTrials.gov Identifier: NCT00985673). GlaxoSmithKline Biologicals SA. also took in charge all costs associated with the development and the publishing of the present manuscript. *Arepanrix and FluLaval* are trade marks of GlaxoSmithKline group of companies.

JML, SH and SM’s institution has received research funding for research studies from GSK, Sanofi Pasteur, Novartis, Dymaxion, Medimmune and Merck. JML, SH and SM have served in volunteer advisory capacities for the Public Health Agency of Canada and the Government of Nova Scotia.

Drs Frenette and Chu are principal investigators in studies funded by GlaxoSmithKline. All participating institutions received compensation for study involvement and travel related to this study. Ping Li and David Vaughn are employees of GlaxoSmithKline group of companies and own stock in the company.

## Authors’ contributions

All authors had full access to the data. The corresponding author had final responsibility to submit for publication. All authors participated in the implementation of the study including substantial contributions to conception and design, the gathering of the data, or analysis and interpretation of the data. All authors were involved in the drafting of the article or revising it critically for important intellectual content, and final approval of the manuscript.

## Pre-publication history

The pre-publication history for this paper can be accessed here:

http://www.biomedcentral.com/1471-2334/12/279/prepub

## Supplementary Material

Additional file 1 **Figure S1.** Haemagglutination inhibition antibodies against vaccine homologous A/California/7/2009 strain [CHMP/CBER criteria] (According To Protocol cohort for immunogenicity). Group definitions: Group A: Group TIV+Plac/15/15: TIV+Placebo (Day 0); 15 μg (Day 21); 15 μg (Day 42); Group B: Group TIV+Plac/AS/AS: TIV+Placebo (Day 0); 3.75 μg/AS03 (Day 21); 3.75 μg/AS03 (Day 42); Group C: Group 15+TIV/15/Plac: 15 μg+TIV (Day 0); 15 μg (Day 21); Placebo (Day 42); Group D: Group AS+TIV/AS/Plac: 3.75 μg/AS03+TIV (Day 0); 3.75 μg/AS03 (Day 21); Placebo (Day 42); Group E: Group 15+Plac/15/TIV: 15 μg+Placebo (Day 0); 15 μg (Day 21); TIV (Day 42); Group F: Group AS+Plac/AS/TIV: 3.75 μg/AS03+Placebo (Day 0); 3.75 μg/AS03 (Day 21); TIV (Day 42); Dotted lines indicate the CHMP/CBER cut-off criteria for HI antibody immune response against pandemic influenza strains in subjects aged 18–60 years (SCR: 40%; SPR: 70%; GMFR: 2.5). Click here for file
