# Supplementary figures and images for: A randomized, controlled non-inferiority trial comparing A(H1N1)pmd09 vaccine antigen, with and without AS03 adjuvant system, co-administered or sequentially administered with an inactivated trivalent seasonal influenza vaccine
Source: BMC Infect Dis. 2012 Oct 30;12:279. doi: 10.1186/1471-2334-12-279 (PMC3529122; doi:10.1186/1471-2334-12-279)

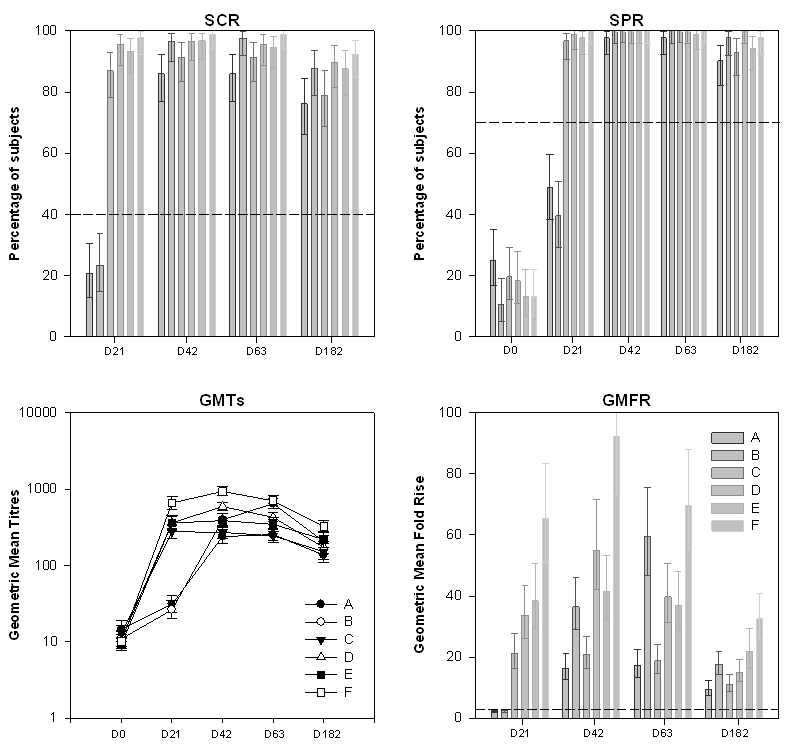

Supplement: Additional file 1 — Figure S1. Haemagglutination inhibition antibodies against vaccine homologous A/California/7/2009 strain [CHMP/CBER criteria] (According To Protocol cohort for immunogenicity). Group definitions: Group A: Group TIV+Plac/15/15: TIV+Placebo (Day 0); 15 μg (Day 21); 15 μg (Day 42); Group B: Group TIV+Plac/AS/AS: TIV+Placebo (Day 0); 3.75 μg/AS03 (Day 21); 3.75 μg/AS03 (Day 42); Group C: Group 15+TIV/15/Plac: 15 μg+TIV (Day 0); 15 μg (Day 21); Placebo (Day 42); Group D: Group AS+TIV/AS/Plac: 3.75 μg/AS03+TIV (Day 0); 3.75 μg/AS03 (Day 21); Placebo (Day 42); Group E: Group 15+Plac/15/TIV: 15 μg+Placebo (Day 0); 15 μg (Day 21); TIV (Day 42); Group F: Group AS+Plac/AS/TIV: 3.75 μg/AS03+Placebo (Day 0); 3.75 μg/AS03 (Day 21); TIV (Day 42); Dotted lines indicate the CHMP/CBER cut-off criteria for HI antibody immune response against pandemic influenza strains in subjects aged 18–60 years (SCR: 40%; SPR: 70%; GMFR: 2.5). [file 1471-2334-12-279-S1.tiff]
